# Strontium ranelate enriched *Ruminococcus albus* in the gut microbiome of Sprague–Dawley rats with postmenopausal osteoporosis

**DOI:** 10.1186/s12866-023-03109-z

**Published:** 2023-11-27

**Authors:** Xiao Xiao, Yuanyuan Cui, Huigai Lu, Jiaqi Wang, Jing Yang, Long Liu, Zhixin Liu, Xiaohong Peng, Hong Cao, Xinghui Liu, Xiuli Wei

**Affiliations:** 1 School of Basic Medicine, Hubei University of Medicine, Shiyan, Hubei PR China; 2 Key Laboratory of Pathogenic Biology, Guilin Medical University, Guilin, Guangxi PR China; 3 Department of Orthopedics, Renmin Hospital, Hubei University of Medicine, Shiyan, Hubei PR China

**Keywords:** Strontium ranelate, Gut microbiome, Postmenopausal osteoporosis, *Ruminococcus*, Lycopene

## Abstract

**Background:**

Gut microbiome is critical to our human health and is related to postmenopausal osteoporosis (PMO). Strontium ranelate (SrR) is an anti-osteoporosis oral drug that can promote osteoblast formation and inhibit osteoclast formation. However, the effect of SrR on gut microbiome has been rarely studied. Therefore, we investigated the effect of oral SrR on gut microbiome and metabolic profiles.

**Results:**

In this study, we used ovariectomized (OVX) Sprague–Dawley rats to construct a PMO model and applied oral SrR for 6 weeks. The relative abundance of intestinal microbiome was investigated by 16S rRNA metagenomic sequencing. Ultra-high-performance liquid chromatography-mass spectrometry (UHPLC-MS) was used to analyze changes in metabolites of intestinal contents. Results demonstrated that 6-week oral SrR alleviated osteoporosis and significantly changed the composition of the gut microbiome and metabolic profiles of OVX rats. *Ruminococcus*, *Akkermansia* and *Oscillospira* were significantly enriched in the gut of OVX rats after 6-week oral SrR. Especially, the species *R. albus* showed the greatest importance by a random forest classifier between OVX and OVX_Sr group. The enrichment of *R. albus* in the gut was positively correlated with bone mineral density and the accumulation of lycopene and glutaric acid, which also significantly elevated after oral SrR.

**Conclusions:**

We discovered that oral SrR can improve bone health while stimulate the accumulation of gut microbe *R. albus* and metabolites (lycopene and glutaric acid). The results suggested possible connections between oral SrR and the gut-bone axis, which may provide new insight into the treatment/prevention of osteoporosis.

**Supplementary Information:**

The online version contains supplementary material available at 10.1186/s12866-023-03109-z.

## Background

Bone is a dynamic organ that relies on the balance between bone formation and bone resorption to maintain normal function [1]. Osteoporosis is a bone disease due to an imbalance of bone remodeling, which causes bone resorption to exceed bone formation and leads to loss of bone density and regression of the bone microstructure [2]. In elder women, the most common risk factor of osteoporosis is the lack of estrogen after menopause. Common treatments recommended for postmenopausal osteoporosis (PMO) include anabolic and antiresorptive therapies, which can either increase osteoblastic bone formation or decrease osteoclastic bone resorption [3, 4]. Although currently approved treatments are safe and generally well tolerated, long-term side effects and high prices remain as challenges [5]. Therefore, further studies on osteoporosis treatment options with minor side effects are necessary.

Strontium ranelate (SrR) has a dual effect on bone turnover, that is, it can stimulate bone formation and decrease bone resorption, thereby preventing bone loss and micro-architecture degradation [6–8]. In clinical trials, oral SrR increased the bone mineral density and reduced the risk of vertebral fractures in patients with PMO [7]. In 2004, SrR has been authorized for PMO with severe osteoporosis in Europe to reduce the risk of vertebral and hip fracture [9]. However, it has a high risk of serious cardiovascular conditions and side effects, such as skin reaction hypersensitivity, diarrhea, nausea, and liver inflammation; as such, SrR has been recommended for patients at high fracture risk who do not have cardiovascular risk factors [9, 10]. Therefore, a method using SrR that can conserve the beneficial effect on osteoporosis and reduce the side effects should be developed.

Gut microbiome is a complex and dynamic microbial community that consists of approximately 100 trillion microbes, including bacteria, virus, fungi, and protozoa [11]. It exists in the human gastrointestinal tract and is now regarded as a vital organ to human health [12, 13]. Osteoporosis is closely associated with the gut microbiome, which can regulate bone metabolism by influencing the immune system, nutrient absorption, and gut permeability [14, 15]. Sjogren et al. [16, 17] found that the bone mass of conventional raised mice was lower than that of germ-free mice, which can be recovered by probiotic supplementation. *Lactococcus* and *Bifidobacterium* can prevent the bone loss of mice caused by estrogen deficiency [18, 19]. In postmenopausal women, 12 months of oral 1 × 10^10^ *L. reuteri* ATCC PTA 6475 every day significantly inhibited bone density reduction [20]. However, the mechanism of the effect of the gut microbiome on osteoporosis is complex and needs further exploration.

The fate and activity of drugs are frequently dictated not only by the host per se but also by microorganisms in the gastrointestinal tract [17]. The interactions between drugs and the gut microbiome may have the potential to enhance drug efficacy [21]. Non-antibiotic oral drugs showed great impact on the gut microbiome, thereby indirectly influencing responses to treatments [22, 23]. SrR is an oral administered drug for osteoporosis and has absolute bio-availability of 27% after a dose of 2 g [24]. Therefore, SrR is inevitably exposed to and interacted with the gut microbiome [25]. However, the relationships between oral SrR, gut microbiome signatures and PMO have not been satisfactorily examined. Therefore, the present study aimed to determine the effect of oral SrR supplementation on gut microbiome composition and metabolism. Ovariectomy (OVX) was conducted on Sprague–Dawley (SD) rats to create PMO model. Oral SrR supplementation was conducted for 6 weeks. We applied an integrated approach of 16S rRNA gene metagenomic sequencing combined with colonic ultra-high-performance liquid chromatography-mass spectrometry (UHPLC-MS) to determine whether specific bacterial genera and metabolites are associated with oral SrR. This study evaluated the interactions between the gut microbiome, PMO, and oral SrR. The results provide new insights into the therapeutic mechanism of oral SrR and the clinical strategy of PMO with less side effects.

## Results

### SrR alleviate osteoporosis of OVX rats

**Fig. 1:**
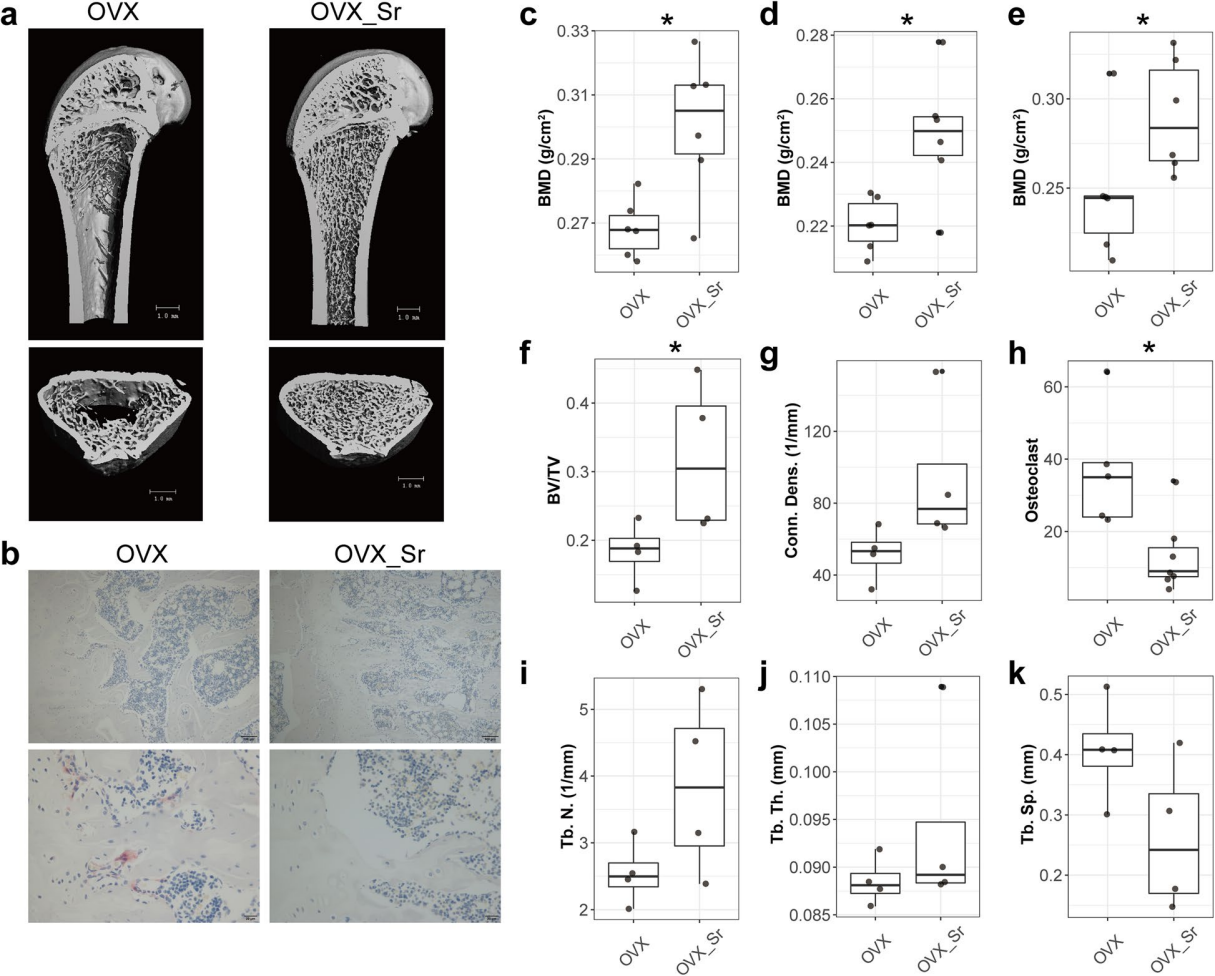
Six-week oral SrR alleviated osteoporosis of OVX rats. **a** Representative reconstruction of trabecular bone for calculation. Scale bar, 1mm. **b** TRAP stain of tibias with 100 × (up) and 400 × (bottom) magnifications. BMD of the whole body (**c**), body without head (**d**), and hindquarters (**e**). BV/TV (**f**), connectivity density (**g**), trabecular number (**i**), trabecular thickness (**j**), and trabecular separation (**k**) on distal femur were analyzed by μCT. **h** Osteoclast cell number per femur surface. The difference between OVX and OVX_Sr groups was analyzed by Wilcoxon rank sum test

Treatment with 6-week oral SrR significantly improved the trabecular structure (Fig. 1a) and increased the bone mineral density (BMD) and bone volume/total volume (BV/TV) of OVX rats (Fig. 1c-f). After 6-week oral SrR, the osteoclast cell number of OVX rats significantly decreased (Fig. 1b and h). Compared with OVX group, the connectivity density (Conn. Dens.), trabecular number (Tb. N.), and trabecular thickness (Tb. Th.) of OVX_Sr rats was elevated but the increase was not significant (Fig. 1g, i and j). The trabecular separation (Tb. Sp.) of OVX_Sr rats decreased, but the effect was not significant (Fig. 1k). Hence, 6-week oral SrR alleviated the osteoporosis of OVX rats.

### Gut microbiome significantly distinguish the OVX_Sr group from the OVX group

**Fig. 2:**
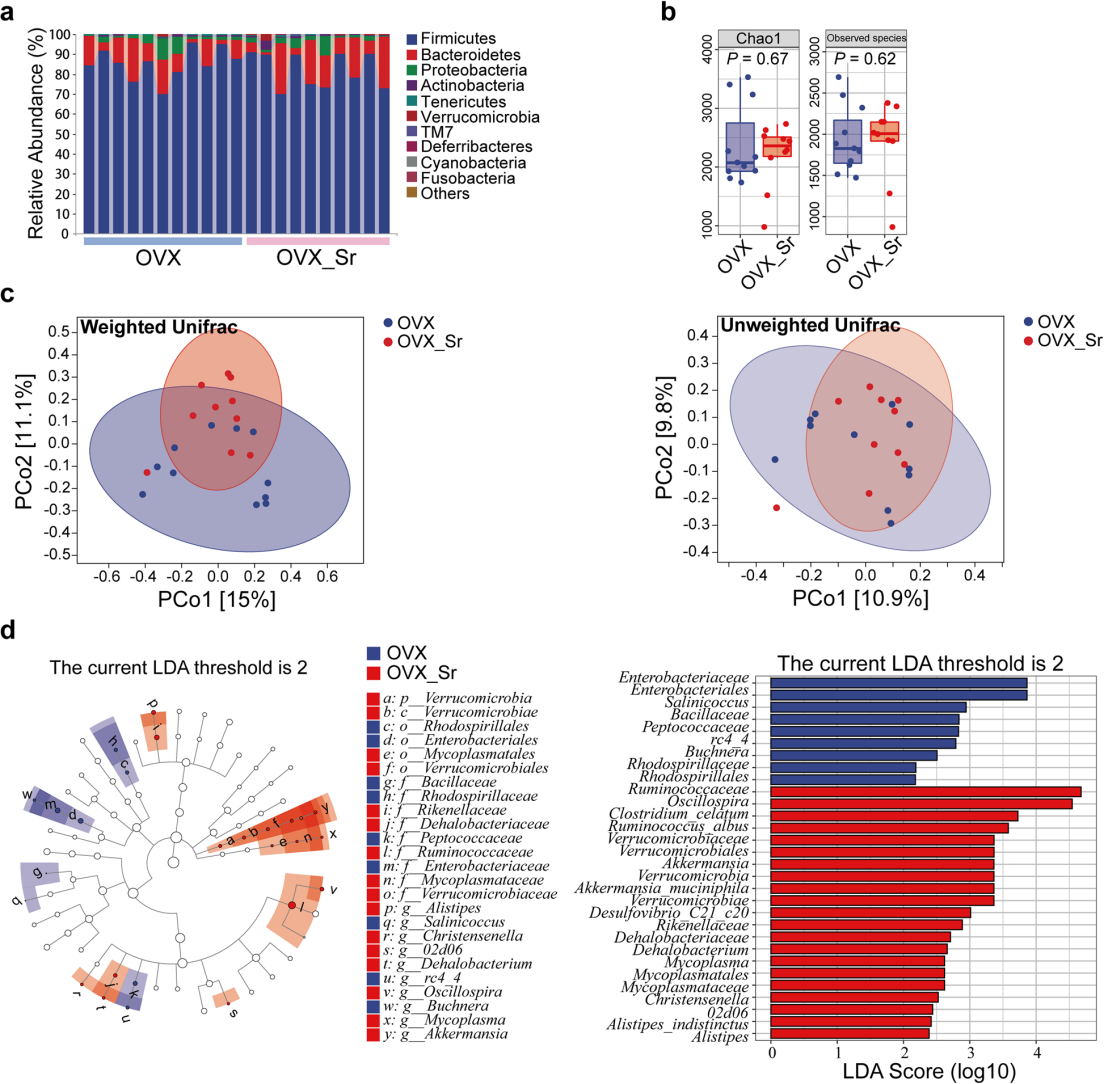
Comparison of the gut microbiome between OVX and OVX_Sr groups. **a** Composition of the gut microbiome at phylum level. **b** Chao 1 and observed species indices showed no significant difference between OVX and OVX_Sr groups. **c** PCoA of the gut microbiome based on WUF and UUF. **d** Significantly changed microbes after 6-week oral SrR determined using LEfSe analyses

After quality control, 840 139 clean reads were obtained in the OVX group (*n* = 11) and OVX_Sr group (*n* = 10). The clean reads generated 5 430 OTUs, including 15 phyla, 30 classes, 52 orders, 99 families and 200 genera. At phylum level, the composition of the gut microbiome in OVX and OVX_Sr rats were mainly consisted of Firmicutes and Bacteroidetes (Fig. 2a), and the difference was not significant as determined by Wilcoxon rank sum test (Supplementary Table 1). The alpha diversity also exhibited no significant difference between OVX and OVX_Sr group (Fig. 2b). Principal coordinate analyses (PCoA) showed that the gut microbiome of OVX and OVX_Sr group were slightly separated along PCo2 based on weighted Unifrac distance (WUF) (Fig. 2c). By employing permutational multivariate analysis of variance (PerMANOVA), the gut microbiome composition significantly differed between OVX and OVX_Sr groups (WUF: F = 2.252, *P* = 0.021; unweighted Unifrac distance (UUF): F = 1.252, *P* = 0.042; Supplementary Table 2). Hence, oral SrR has significant effect on the beta diversity of the gut microbiome in OVX rats.

**Fig. 3:**
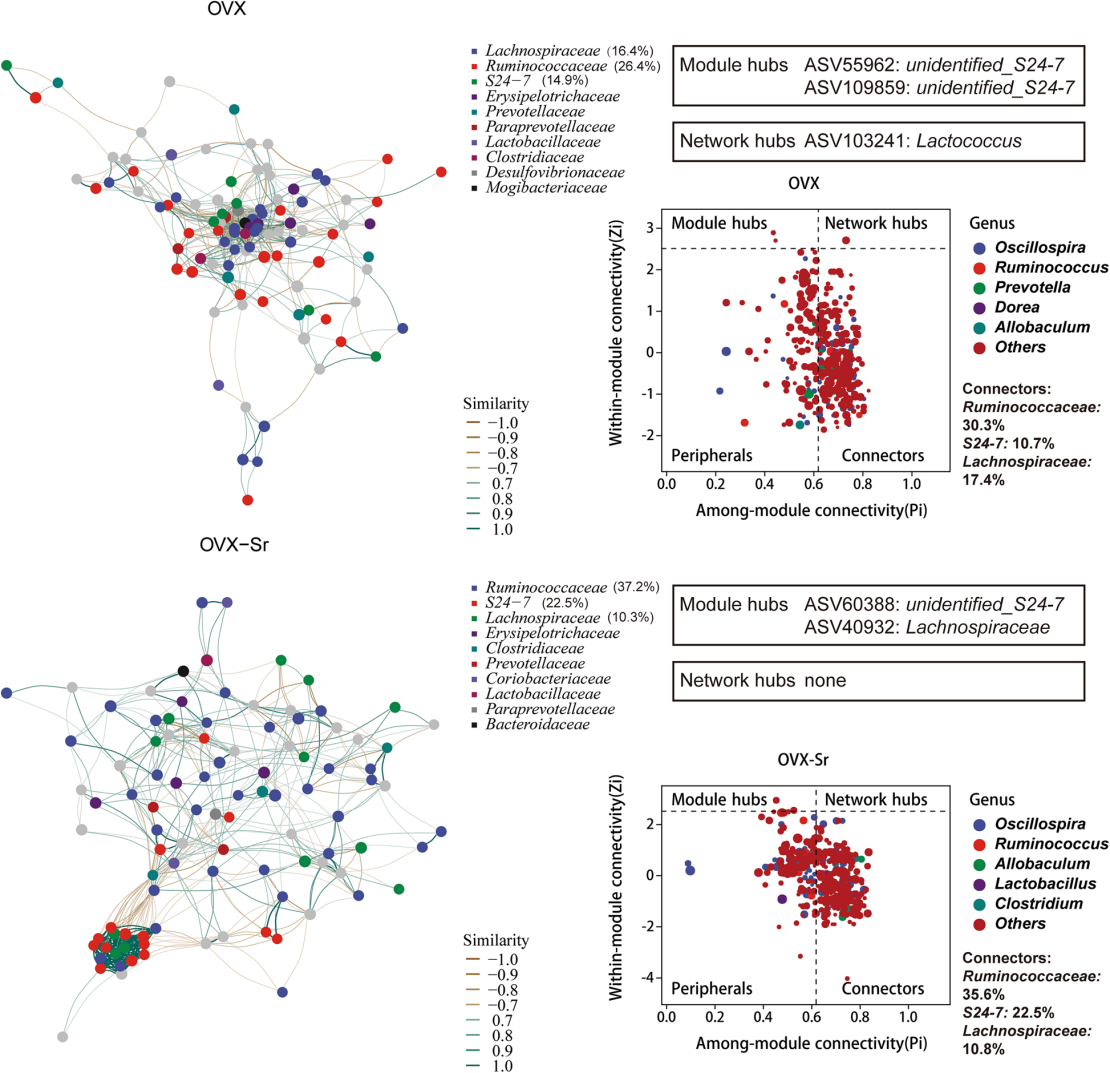
Networks and Zi-Pi plots of OVX and OVX_Sr gut microbiome based on SparCC correlation coefficients. Each dot represents each ASV. Different colors of dots represent different family (network visualization) or genus (Zi-Pi plots). Different colors of lines represent different correlation coefficients. The role of nodes was characterized by within-module connectivity (Zi) and among-module connectivity (Pi). Peripheral nodes (Zi ≤ 2.5, Pi ≤ 0.62), connectors (Zi ≤ 2.5, Pi > 0.62), module hubs (Zi > 2.5, Pi ≤ 0.62), and network hubs (Zi > 2.5, Pi > 0.62) are shown in the Zi-Pi plots

To further identify the variation of gut microbiome triggered by oral SrR, LDA effect size (LEfSe) analysis was employed to determine differences between OVX and OVX_Sr groups. The relative abundance of *Oscillospira*, *Clostridium celatum*, *Ruminococcus albus*, and *Akkermansia muciniphila* significantly increased in the OVX_Sr group compared with that in the OVX group (Fig. 2d). At OTU level, networks were constructed in OVX and OVX_Sr groups based on Spearman’s relationships separately (│r│ > 0.7, *P* < 0.05, Fig. 3). Among-module connectivity (Pi) and within-module connectivity (Zi) of each node were calculated. The topological roles of all nodes were categorized into four types: peripherals (Zi ≤ 2.5, Pi ≤ 0.62), connectors (Zi ≤ 2.5, Pi > 0.62), module hubs (Zi > 2.5, Pi ≤ 0.62) and network hubs (Zi > 2.5, Pi > 0.62). In the OVX group, the nodes of the network mainly belonged to Ruminococcaceae (26.4%), Lachnospiraceae (16.4%) and S24-7 (14.9%). Ruminococcaceae accounted for 30.3% of all the connectors. In the OVX_Sr group, the nodes of the network mainly belonged to Ruminococcaceae (37.2%), S24-7 (22.5%) and Lachnospiraceae (10.3%). The proportions of Ruminococcaceae and S24-7 in the OVX_Sr group were higher than those in the OVX group, and the proportion of Lachnospiraceae declined. Ruminococcaceae accounted for 35.6% of all the connectors in the OVX_Sr group, which was higher than that in the OVX group. The results suggested that Ruminococcaceae played a more important role in the OVX_Sr gut microbiome than in the OVX group.

### Metabolomics analysis of colonic samples

**Fig. 4:**
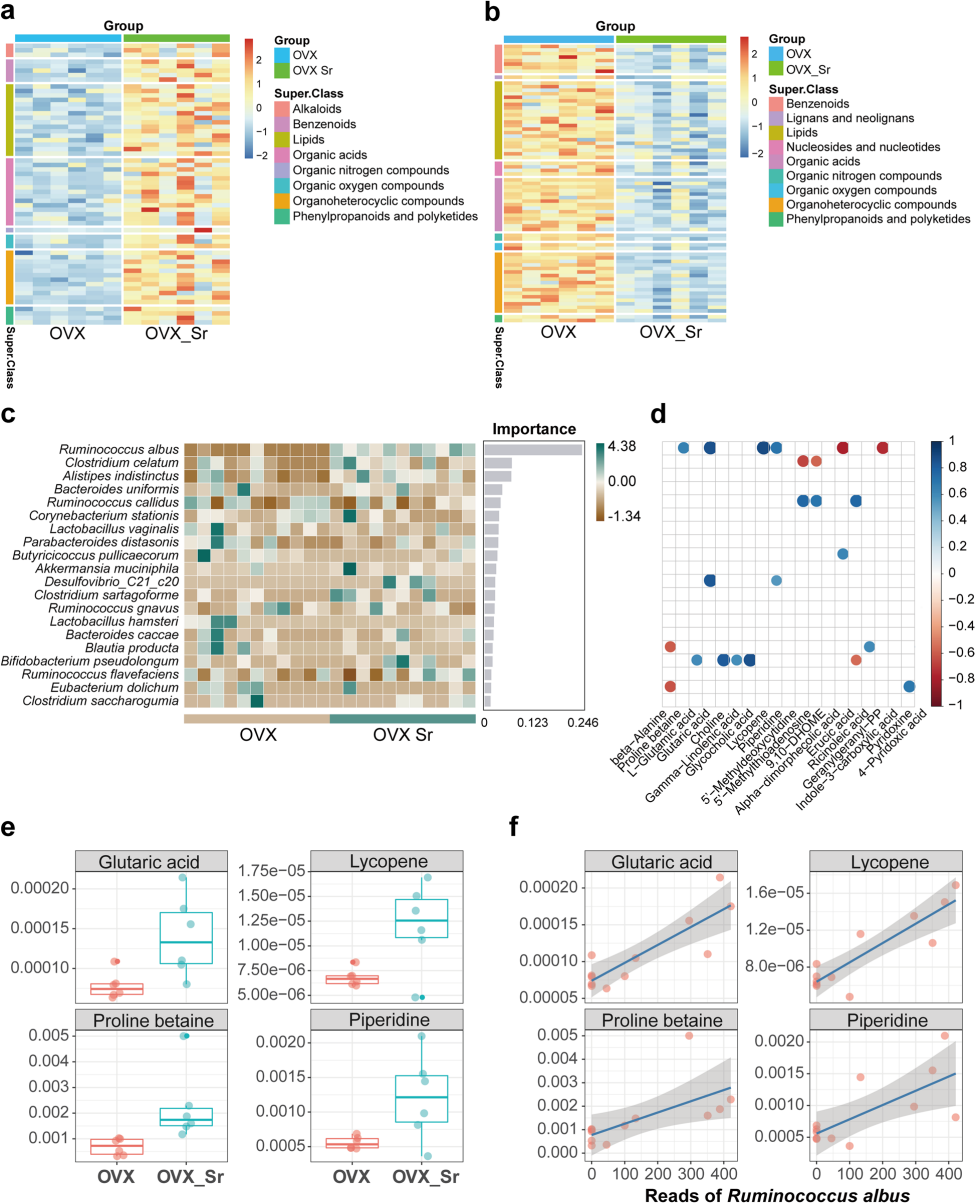
Gut metabolites enriched in OVX_Sr group (**a**) and OVX group (**b**). Different row-side colors represent different super classes into which metabolites were classified. Different column-side colors represent different groups. **c**The key stone species determined by a random forest classifier. **d** Spearman’s relationships between the key stone species and the metabolites. Each dot represents significant relationship (*P* < 0.05). The accumulation of four metabolites (**e**) in OVX_Sr increased with increased abundance of *R. albus* (**f**). Differences in metabolites between OVX and OVX_Sr groups (E) were calculated by using Wilcoxon rank sum tests

To further explore the potential contributing metabolites, UHPLC-MS was employed to analyze the gut metabolites of OVX and OVX_Sr rats. The gut metabolites mainly included alkaloids, benzenoids, lipids, organic acids, organic nitrogen compounds, organic oxygen compounds, organoheterocyclic compounds, phenylpropanoids and polyketides. The result of the principal component analysis (PCA) exhibited great differences in the metabolite profiles between OVX and OVX_Sr groups (Supplementary Fig. 1). The variable importance for the projection (VIP) of orthogonal projections to latent structure discriminant analysis (OPLS-DA) was calculated to select significantly changed metabolites between OVX and OVX_Sr groups (VIP > 1.0 and *P* < 0.05; Fig. 4a and b, Supplementary Tables 3 and 4). Among the significantly changed metabolites, the majority were lipids, organic acids and organoheterocyclic compounds. Compounds with the highest fragmentation score based on MS2 library searches mainly included beta-alanine, proline betaine, L-glutamic acid, glutaric acid, choline, gamma-linolenic acid, glycocholic acid, lycopene, piperidine and 5’-methyldeoxycytidine.

We applied a random forest classifier to the core sequence clusters of OVX and OVX_Sr groups to determine the key stone species (Fig. 4c). *R. albus* showed the biggest importance and had higher relative abundance in the OVX_Sr group than in the OVX group. The relative abundance of *R. albus* showed significantly positive relationships to significantly enriched metabolites after oral SrR (proline betaine, glutaric acid, lycopene and piperidine), and negative relationships to erucic acid and indole-3-carboxylic acid (Fig. 4d and e). These results were also confirmed by linear regression shown in Fig. 4f. Hence, the elevation of *R. albus* was accompanied by the accumulation of proline betaine, glutaric acid, lycopene and piperidine in the gut after 6-week oral SrR. These accumulations may be related to the relief of osteoporosis symptoms.

## Discussion

In the present study, we verified that oral SrR increased the BMD and improved the trabecular microstructure of OVX rats [26]. Meanwhile, oral SrR significantly modified the gut microbiome and its metabolic activity. Therefore, we suspect possible associations among oral SrR, gut microbiome, and bone health.

*Ruminococcus* species are critical members of the rumen microbial community with cellulolytic and short chain fatty acids (SCFAs)-producing ability as potential probiotics [27, 28]. Research on the gut–bone axis detected higher abundance of *Ruminococcus* in healthy individuals compared with patients with osteoporosis [29, 30]. In this study, the genus *Ruminococcus*, especially *R. albus*, was enriched after 6-week of oral SrR in OVX rats (Supplementary Fig. 2a). Moreover, the relative abundance of *R. albus* positively correlated with BMD (Supplementary Fig. 2c and Supplementary Table 5). *R. albus* is a promising candidate for probiotics in human gut [31]. It has been found that heat-killed *R. albus* can protect neurons from oxidative damage [32]. Hence, we speculated that *Ruminococcus*, especially *R. albus*, may be associated with osteoporosis alleviation.

Furthermore, we found that *R. albus* was significantly correlated with four kinds of metabolites, namely, proline betaine, glutaric acid, lycopene, and piperidine (Fig. 4). These compounds were also enriched in the gut by oral SrR (Fig. 4 and Supplementary Table 3). Lycopene can promote osteoblast proliferation and differentiation in vitro. On the other hand, it can inhibit mineral resorption by inhibiting osteoclast formation and ROS production in vitro [33–35]. In vivo, several studies verified that the supplementation of lycopene can significantly decrease oxidative stress parameters and bone resorption [36, 37]. Glutaric acid is one of the SCFAs produced by bacterial fermentation of non-digestible carbohydrates [38]. SCFAs can affect local and systemic immune functions, and act as potent regulators of osteoclasts to prevent postmenopausal and inflammation-induced bone loss [39, 40]. Consistent with the trend in this study, Cabrera et al. found that glutaric acid in sheep plasma decreased one month after OVX [38]. Therefore, we speculated that the enrichment of *R. albus*, lycopene and glutaric acid in the gut by oral SrR may contribute to the relief of osteoporosis of OVX rats.

The abundance of *Akkermansia* and *Oscillospira* also significantly increased in the OVX_Sr group compared with that in the OVX group (Fig. 2 and Supplementary Fig. 2b). *Akkermansia* is a potential probiotic that can participate in host immune regulation and promote intestinal health [41, 42]. *A. muciniphila* decreased in individuals with osteoporosis and osteopenia compared with that in healthy ones, which suggested the potential relationship between *A. muciniphila* and bone health [43, 44]. *Oscillospira* is commonly detected in the human gut by metagenomic sequencing. Although *Oscillospira* is rarely cultivated, it has been regarded as a promising probiotic for inflammatory bowel diseases and leanness in humans [45, 46]. Further, the gut microbiome is a dynamic and complicated whole, and the symbiosis between the gut microbiome and the host requires a delicate balance. Therefore, *Akkermansia* and *Oscillospira* may cooperate with *Ruminococcus* in functioning in osteoporosis. Further, this study was conducted using 16S rRNA metagenome sequencing. The resolution and accuracy may be lower than metagenomic sequencing and culture-dependent methods. Therefore, our conclusion still needs confirmation by further experiments on isolated *R. albus* strain.

For recent researches mainly focus on the regulation of gut microbiome on bone homeostasis, less attention has been paid to the influence of bone mass on gut microbiome. Although several studies found that postmenopausal women with different BMDs had different gut microbiome compositions, it is hard to define the causal relationship between BMD and gut microbiome [47, 48]. There seems to be no conclusive evidence on whether bone mass can influence gut flora [49]. It has been found that proton pump inhibitors can indirectly influence gut microbiome by reducing the acidity barrier of the stomach, which allows oral microbes pass through the stomach to the gut [23]. The direct influence of drugs might be the main mechanism for modulating gut microbiome [23]. Non-antibiotic drugs can directly influence gut microenvironment and modulate the growth of gut bacteria and bacterial native metabolism [50]. We speculated that the direct effects of SrR on the gut microbiome might play a leading role, because bones can neither directly contact with outer environment nor be inhabited by diverse microbes normally. However, we cannot rule out the possibility of indirectly influence of SrR through other organ systems. Further validation analysis is to investigate the influence of SrR on the gut microbiome of sham operation rats, and verify the direct effect of SrR on the growth and metabolism of gut bacteria in vitro, which may contribute to validate the causal relationship and mechanism on PMO and gut microbiome.

## Conclusion

In conclusion, we have shown that oral SrR alleviated osteoporosis and significantly changed the composition of the gut microbiome and metabolic profiles of OVX rats. Several gut microbes and metabolites significantly enriched after oral SrR. Furthermore, positive relationship between specific gut microbe and BMD was detected, which indicating the potential function of gut microbiome during the treatment of osteoporosis. Our findings may provide new insights in the treatment/prevention of osteoporosis through gut–bone axis.

## Methods

### Animals and sample collection

We purchased 30 24-week-old SD female rats from the Laboratory Animal Center (Hubei University of Medicine). They were raised without specific pathogens. The rats were fed sterile food and given autoclaved water ad libitum. During the experimental period, the mice were housed in an animal room under controlled environmental conditions at a temperature of 22 ± 2 °C, relative humidity of 45 ± 5%, and a 12 h light/dark cycle. After feeding for one week under this condition, the rats were randomly divided into two groups including a OVX group (*n* = 22) and a Sham group (*n* = 8). The OVX group was subjected to ovariectomy to construct PMO models. The Sham group was subjected to sham operation to remove equal volume of fat near the ovaries. After 6 weeks, BMD of 7 individuals from Sham group and 8 individuals which were randomly selected from OVX group was examined to validate the model (Supplementary Fig. 3).

After establishing the model, we randomly divided the OVX group into OVX_Sr group (*n* = 10) and OVX group (*n* = 11). SrR (Sigma–Aldrich Trading Co. Ltd., Shanghai, China) was added to the chow for OVX_Sr group at a concentration of 650 mg/kg body weight/day for 6 weeks. The OVX were treated with normal chow for 6 weeks.

After the experimental period, all rats were sacrificed by over-anesthesia with pentobarbital sodium (intraperitoneal injection at a dose of 150 mg/kg). Bone mineral content, microstructure, and histomorphology of randomly selected rats from each group were assessed. Colonic contents were collected and stored at –80 °C for further analysis. All animal experiments were performed in accordance with relevant guidelines and regulations, and were approved by the Animal Care and Use Committee at Hubei University of Medicine (No. 2019–089). The experimental procedure was provided as Supplementary Fig. 4. The weight of animal at different time points was provided as Supplementary Fig. 5.

### Micro CT

Micro-CT (μCT50 in vitro scanner; Scanco Medical, AG, Switzerland) was used to assess femur microstructure in vitro. The femoral trabecular structure was initially scanned at the level of the growth plate and extended 50 slices. From this region, 100 slices were chosen for the evaluation. The femoral cortical structure was assessed through 100 continuous CT slides from the bone midshaft. Quantitative parameters evaluated were bone volume/total volume (BV/TV), connectivity density (Conn. Dens., 1/mm), trabecular number (Tb. N., 1/mm), trabecular thickness (Tb. Th., mm), and trabecular separation (Tb. Sp., mm).

### Bone mineral content measurement

The BMD of the rats were measured by dual-energy X-ray absorptiometry (DXA) (Hologic Discovery A, San Diego, USA). The rats were positioned on a DXA table in the prone position to scan proper area (whole body, body without head, and hindquarters). Analyses were performed using Apex 4.5.3 software for small animals carried by Hologic Discovery A (San Diego, USA).

### Bone histomorphometry

Tibias were fixed in 4% paraformaldehyde for 72 h and decalcified in EDTA decalcification fluid (Servicebio, Wuhan, China). The decalcified tibias were dehydrated and defatted with graded ethanol (50%–100%) and xylene and then embedded in paraffin [51]. Sections of 4-μm thickness were used for tartrate-resistant acid phosphatase (TRAP) staining following the manufacturer’s instructions of the TRAP dye solution kit (Servicebio, Wuhan, China). All images were taken on an Olympus BX53 microscope with an Olympus DP73 camera (Olympus, Tokyo, Japan) by using Olympus cellSens standard software. Osteoclast number per bone surface was measured. Osteoclasts were identified as TRAP-positive cells with more than three nuclei.

### Amplicon Sequence Variant (ASV)-based analysis of 16S rDNA sequencing data

Total genomic DNA samples were extracted from colonic samples using the Mag-bind soil DNA kit (M5635-02) (Omega Bio-Tek, Norcross, GA, USA) following the manufacturer’s instructions. DNA quantity and quality were measured using a NanoDrop NC2000 spectrophotometer (Thermo Fisher Scientific, MA, USA) and agarose gel electrophoresis, respectively. The V3–V4 region of the bacterial 16S rRNA gene was amplified through PCR using the forward primer 338F (5'-ACTCCTACGGGAGGCAGCA-3') and the reverse primer 806R (5'-GGACTACHVGGGTWTCTAAT-3'). The PCR mixture contained 5 μl of buffer (5 ×), 0.25 μl of Fast pfu DNA Polymerase (5 U/μl), 2 μl (2.5 mM) of dNTPs, 1 μl (10 μM) of each forward and reverse primer, 1 μl of DNA template, and 14.75 μl of ddH_2_O. Thermal cycling consisted of initial denaturation at 98 °C for 5 min, followed by 25 cycles consisting of denaturation at 98 °C for 30 s, annealing at 53 °C for 30 s, and extension at 72 °C for 45 s, with a final extension of 5 min at 72 °C. The PCR amplicons were purified with Vazyme VAHTSTM DNA Clean Beads (Vazyme, Nanjing, China) and quantified using the Quant-iT PicoGreen dsDNA Assay Kit (Invitrogen, CA, USA). The amplicons were pooled in equal amounts. Pair-end 2 × 250 bp sequencing was performed using the Illumina NovaSeq 6000 platform at Shanghai Personalbio Biotechnology Co., Ltd. (Shanghai, China).

Microbiome bioinformatics were performed with QIIME2 2019.4 [52]. Raw sequence data were demultiplexed using the demux plugin followed by primer cutting with cutadapt plugin [53]. The sequences were then quality filtered, denoised, merged and chimera removed using the DADA2 plugin [54]. Non-singleton amplicon sequence variants (ASVs) were aligned with mafft [55] and used to construct a phylogeny with fasttree2 [56]. Alpha diversity and beta diversity (WUF and UUF) were estimated using the diversity plugin with reads rarefied to 26 847 sequences per sample. Taxonomy was assigned to ASVs by using the classify-sklearn naïve Bayes taxonomy classifier in feature–classifier plugin [57] against the Greengenes Database [58].

### Non-target metabolomic analysis

The method we used for detecting gut metabolites was non-target metabolomic analysis. We thawed the colonic content samples (randomly selected 6 samples from each group) on ice. All samples were analyzed individually not pooled. We added 100 mg of the sample to precooled 50% methanol and mixed thoroughly by vortexing. The samples were then incubated on ice for 5 min and centrifuged (15 000 g) at 4 °C for 15 min. The supernatant was stored at -80 °C until subsequent analysis.

We used a Vanquish UHPLC system (Thermo Fisher, 100 mm × 2.1 mm, 1.9 mm) for the chromatographic separation of the samples at a constant temperature of 40 °C and an Orbitrap Q Exactive series mass spectrometer (Thermo Fisher) to detect eluted metabolites. C18 column was used in the UHPLC/MS analysis. The sample injection volume was 5 ml, and the column flow rate was maintained at 0.2 ml/min. The mobile phase contained two solvent eluents. In positive mode, eluent A was 0.1% (v/v) formic acid in water, and eluent B was methanol; in negative mode, eluent A was 5 mM ammonium acetate with a pH of 9.0, and eluent B was methanol. The gradient elution was 2% B for 1.5 min, 2–100% B for 12.0 min, 100% B for 14.0 min, 100–2% B for 14.1 min, and 2% B for 17 min. To analyze the samples, we set the mass spectrometer spray voltage to 3.2 kV, the capillary temperature to 320 °C, the sheath gas flow rate to 35 arb, and the auxiliary gas flow rate to 10 arb.

### Statistical analysis

The gut microbiome was analyzed on the normalized data set. Alpha diversity index and PerMANOVA were calculated or performed with 999 permutations using the ‘Vegan 2.5–7’ package on the R platform (4.0.4) [59]. PCoA based on WUF and UUF distances were implemented by using the R package “ape 5.5” [60]. PCA among groups were performed by using the R package “ade4 1.7–18”. Wilcoxon rank sum tests and linear fitting were conducted with the R package “stats 4.0.4”. A correlation plot was calculated and visualized by using the R package “Hmisc 4.5–0”. Heatmaps were visualized by using R package “pheatmap 1.0.12”. Other figures were generated with the R package “ggplot2 3.3.5”. The ASVs (total reads < 10 or sample number < 5) were filtered before network analyses. Network analyses were performed on the basis of SparCC correlation coefficients (*P* < 0.05) [61]. Topology indices were calculated by using R package “igraph 1.2.6” [62]. The results were visualized by Cytoscape 3.7.1 software [63]. LEfSe analyses and visualizations were performed according to previous study [64].

### Supplementary Information

**Additional file 1: Supplementary figure 1.** PCA of gut metabolic profiles. **Supplementary figure 2.** Significantly changed species (A) and genera (B) between OVX and OVX_Sr group. **Supplementary figure 3.** Bone mineral density (BMD) of OVX and Sham group to validate the PMO model. **Supplementary figure 4.** Flow chart of the experiment. **Supplementary figure 5.** Comparison of animal’s weight at different time points by using Wilcoxn rank sum test. **Table S1.** Wilcoxon rank sum tests of gut microbes between OVX and OVX_Sr group at phylum level. **Table S2.** The PerMANOVA analysis of OVX and OVX_Sr group based on WUF and UUF. **Table S3.** The gut metabolites significantly elevated after one-month of oral SrR. **Table S4.** The gut metabolites significantly decreased after one-month of oral SrR. **Table S5.** The Spearman’s relationship between the relative abundance of *R*. *albus* and BMD. 

## Data Availability

The dataset generated and analyzed in the current study is publicly available in NCBI’s Sequence Read Archive (SRA) repository under the BioProject PRJNA842212 (https://www.ncbi.nlm.nih.gov/bioproject/PRJNA842212; SRA accession ID: SRR19392962-SRR19392969, SRR19392971-SRR19392980, SRR19392982-SRR19392984).

## Abbreviations

PMO: Postmenopausal Osteoporosis
SrR: Strontium Ranelate
OVX: Ovariectomized
SD: Sprague–Dawley
UHPLC-MS: Ultra-High-Performance Liquid Chromatography-Mass Spectrometry
BMD: Bone mineral density
BV/TV: Bone volume/total volume
Conn. Dens.: Connectivity Density
Tb. N.: Trabecular Number
Tb. Th.: Trabecular Thickness
Tb. Sp.: Trabecular Separation
PCoA: Principal Coordinates Analysis
QIIME: Quantitative Insights into Microbial Ecology
WUF: Weighted Unifrac distance
UUF: Unweighted Unifrac distance
ASV: Amplicon Sequence Variant
PerMANOVA: Permutational Analysis of Variance
LEfSe: Linear Discriminant Analysis Effect Size
PCA: Principal Component Analysis
VIP: Variable Importance for The Projection
OPLS-DA: Orthogonal Projections to Latent Structure Discriminant Analysis
SCFA: Short Chain Fatty Acid
TRAP: Tartrate-Resistant Acid Phosphatase
DXA: Dual-Energy X-Ray Absorptiometry
