# Supplementary material for: Strontium ranelate enriched Ruminococcus albus in the gut microbiome of Sprague–Dawley rats with postmenopausal osteoporosis
Source: BMC Microbiol. 2023 Nov 27;23:365. doi: 10.1186/s12866-023-03109-z (PMC10680188; doi:10.1186/s12866-023-03109-z)
Supplement: Supplementary file 1 — Additional file 1: Supplementary figure 1. PCA of gut metabolic profiles. Supplementary figure 2. Significantly changed species (A) and genera (B) between OVX and OVX_Sr group. Supplementary figure 3. Bone mineral density (BMD) of OVX and Sham group to validate the PMO model. Supplementary figure 4. Flow chart of the experiment. Supplementary figure 5. Comparison of animal’s weight at different time points by using Wilcoxn rank sum test. Table S1. Wilcoxon rank sum tests of gut microbes between OVX and OVX_Sr group at phylum level. Table S2. The PerMANOVA analysis of OVX and OVX_Sr group based on WUF and UUF. Table S3. The gut metabolites significantly elevated after one-month of oral SrR. Table S4. The gut metabolites significantly decreased after one-month of oral SrR. Table S5. The Spearman’s relationship between the relative abundance of R. albus and BMD. [file 12866_2023_3109_MOESM1_ESM.pdf]

Supplementary Material

Supplementary Figures and Tables

1.1 Supplementary Figures

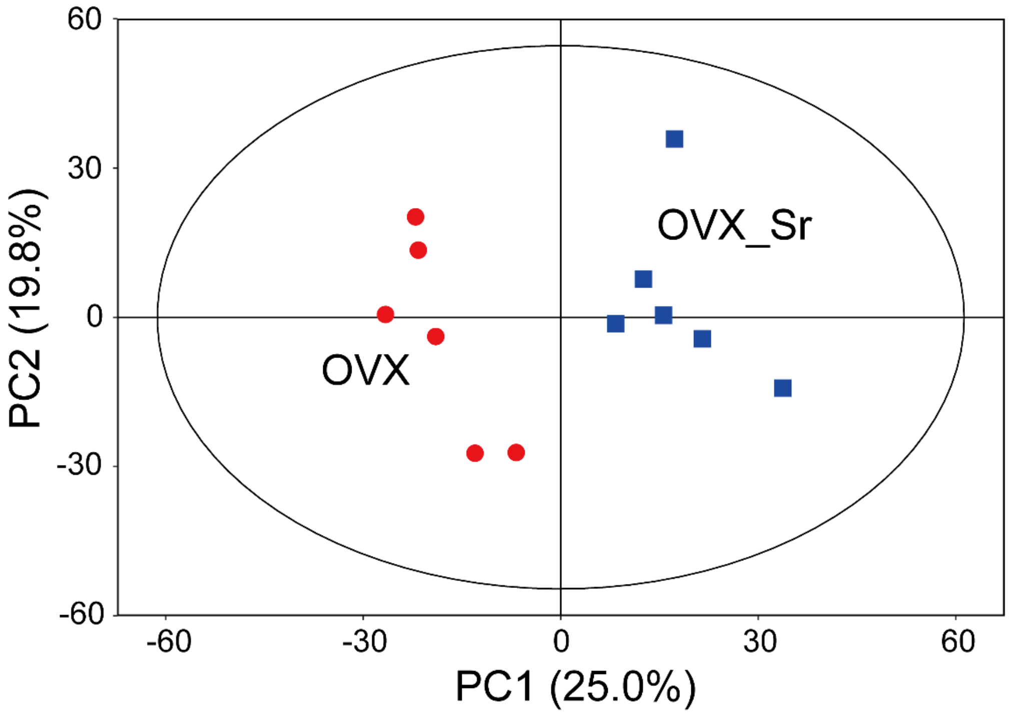

Supplementary figure 1. PCA of gut metabolic profiles.

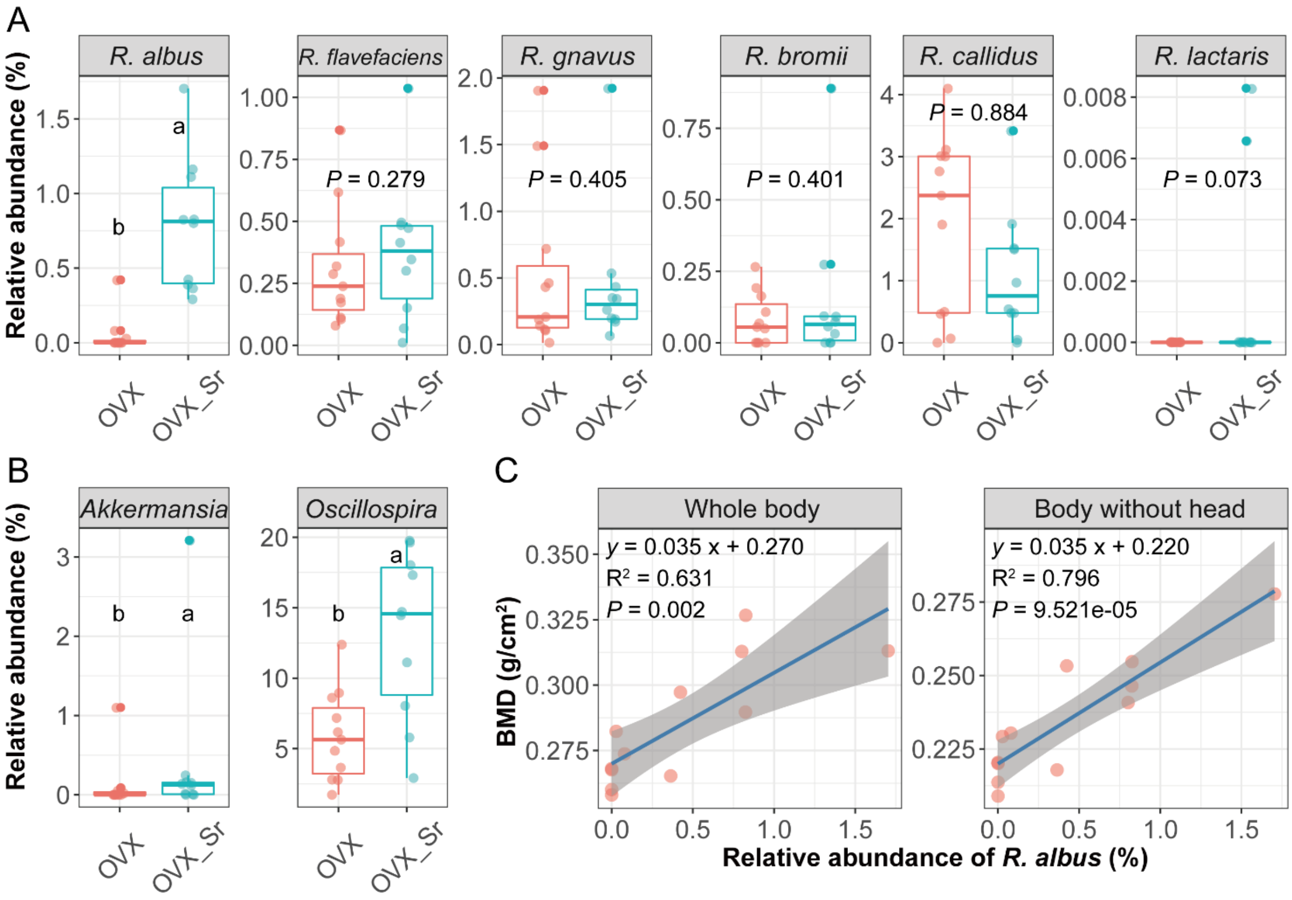

Supplementary figure 2. Significantly changed species (A) and genera (B) between OVX and OVX_Sr group. The differences between OVX and OVX_Sr group were examined by Wilcoxon rank sum test. Different alphabets represent significant difference ($P < 0.05$). **The linear regressions of the relative abundance of *R. albus* and the BMD (C).**

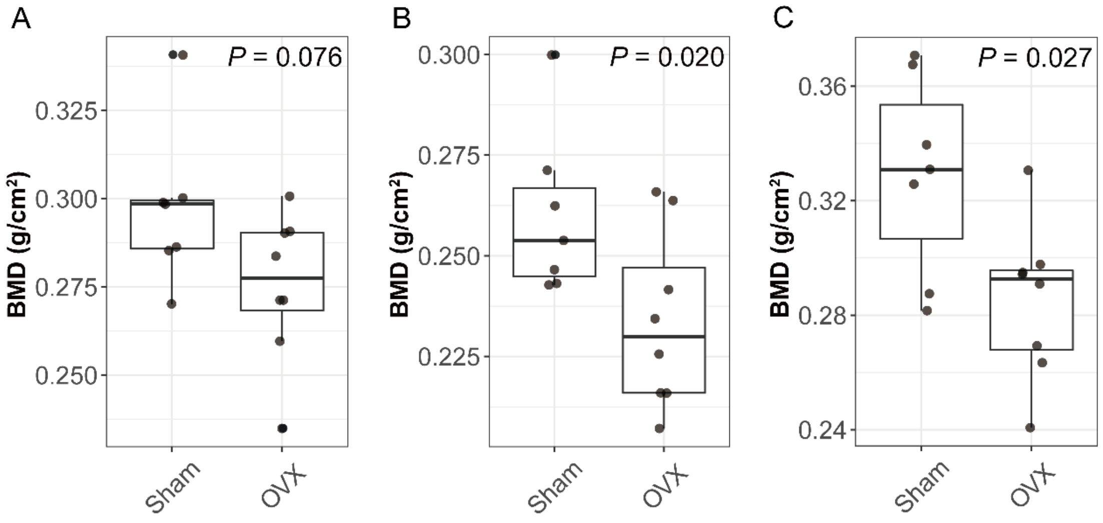

Supplementary figure 3. Bone mineral density (BMD) of OVX and Sham group to validate the PMO model. (A) the BMD of whole body; (B) the BMD of body without the head; (C) the BMD of hindquarters. The differences between OVX and Sham group were examined by Wilcoxon rank sum test.

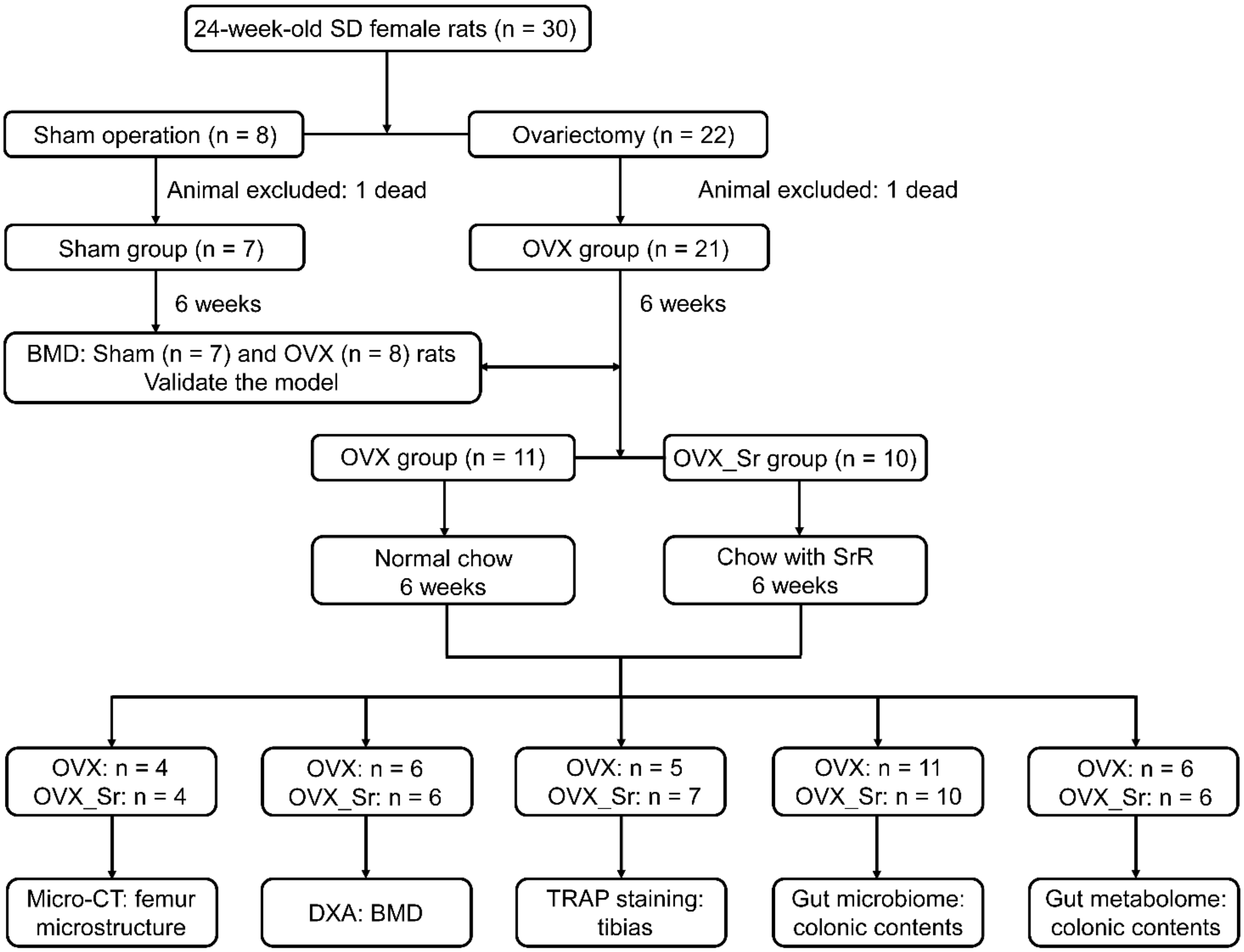

Supplementary figure 4. Flow chart of the experiment.

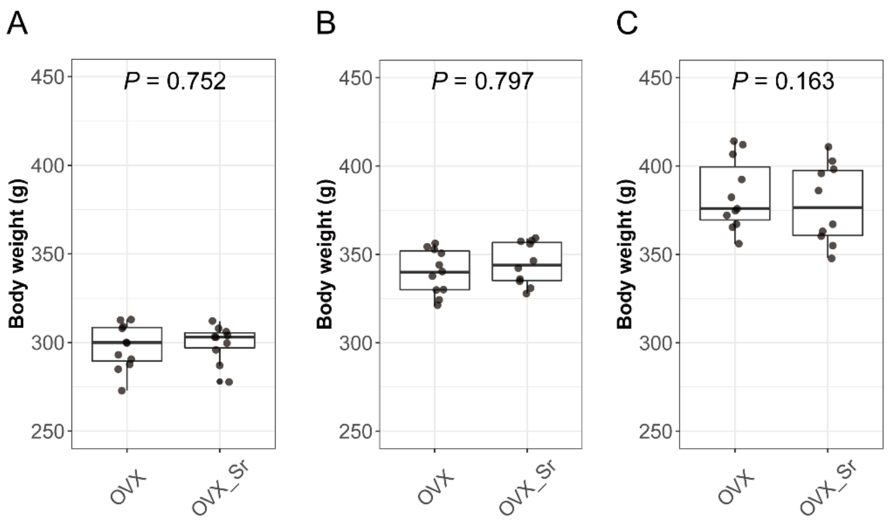

Supplementary figure 5. Comparison of animal's weight at different time points by using Wilcoxn rank sum test. (A) the beginning of experiment; (B) the beginning of SrR treatment; (C) the time of sacrifice.

1.2 Supplementary Tables

Table S1. Wilcoxon rank sum tests of gut microbes between OVX and OVX_Sr group at phylum level.

Phylum	*P* value	Mean of OVX (%)	Mean of OVX_Sr (%)
Firmicutes	0.255781	0.853315	0.819631
Bacteroidetes	0.721368	0.104521	0.134565
Proteobacteria	0.486269	0.031858	0.02823
Tenericutes	0.302365	0.00273	0.002649
Actinobacteria	0.568367	0.005809	0.009267
TM7	0.098558	0.000444	0.001049
Cyanobacteria	0.091824	3.04E-05	0.000132

Table S2. The PerMANOVA analysis of OVX and OVX_Sr group based on WUF and UUF.

WUF	Df	Sum Of Sqs	R^2	F	*P*	
Model	1	0.2985	0.10596	2.2519	0.021	*
Residual	19	2.5185	0.89404			
Total	20	2.817	1			
UUF	Df	Sum Of Sqs	R^2	F	*P*	
Model	1	0.2889	0.06181	1.2517	0.042	*
Residual	19	4.386	0.93819			
Total	20	4.6749	1			

The number of permutations is 999. Significant codes: $P < 0.05$: '*'; $P < 0.01$: '**'; $P < 0.001$: '***'.

Table S3. The gut metabolites significantly elevated after one-month of oral SrR.

Name	Super Class	Mean of OVX	Mean of OVX_Sr	VIP	*P* Value	Fold change	MS2 score
Proline betaine	Organic acids and derivatives	6.93E-04	2.24E-03	1.906	0.043	0.31	0.997
Choline	Organic nitrogen compounds	1.70E-03	1.20E-02	2.179	0.050	0.141	0.997
Glutaric acid	Organic acids and derivatives	7.81E-05	1.40E-04	1.541	0.029	0.558	0.994
Piperidine	Organoheterocyclic compounds	5.56E-04	1.21E-03	1.569	0.048	0.46	0.992
Gamma-Linolenic acid	Lipids and lipid-like molecules	1.09E-03	2.31E-03	1.902	0.007	0.47	0.988
beta-Alanine	Organic acids and derivatives	4.52E-04	8.30E-04	1.584	0.037	0.545	0.979
Glycocholic acid	Lipids and lipid-like molecules	3.00E-06	1.14E-05	1.257	0.041	0.264	0.965
L-Glutamic acid	Organic acids and derivatives	2.21E-04	4.30E-04	1.793	0.026	0.513	0.918
Lycopene	Lipids and lipid-like molecules	6.79E-06	1.21E-05	1.606	0.028	0.562	0.906
Genomorphine	Alkaloids and derivatives	5.01E-05	1.23E-04	1.469	0.026	0.408	0.867
18-Nor-4(19),8,11,13-abietatetraene	Lipids and lipid-like molecules	6.67E-05	1.15E-04	1.509	0.027	0.58	0.86
7,8-Dihydrovomifoliol 9-[rhamnosyl-(1->6)-glucoside]	Lipids and lipid-like molecules	4.26E-05	1.07E-04	2.093	<0.001	0.398	0.86
Isovalerylsarcosine	Organic acids and derivatives	4.59E-05	7.14E-05	1.644	0.023	0.643	0.859
Taccagenin	Lipids and lipid-like molecules	4.39E-06	1.56E-05	1.718	0.013	0.282	0.845
Asparenyol	Benzenoids	1.43E-05	3.42E-05	1.076	0.003	0.417	0.834
Methyl (9Z)-8'-oxo-6,8'-diapo-6-carotenoate	Lipids and lipid-like molecules	1.90E-05	9.52E-05	1.835	0.003	0.2	0.826
6,7-Dihydro-2-methyl-5H-cyclopenta[b]pyrazine	Organoheterocyclic compounds	2.56E-05	3.68E-05	1.463	0.034	0.694	0.826
8-Hydroxycarteolol	Organoheterocyclic compounds	6.12E-06	1.29E-05	1.753	0.012	0.476	0.818
5a-Cholesta-8,24-dien-3-one	Lipids and lipid-like molecules	4.36E-05	7.87E-05	1.541	0.040	0.553	0.804
N'-nitrosonornicotine	Organoheterocyclic compounds	1.63E-04	3.36E-04	1.769	0.008	0.484	0.799

3-Acetoxy-3-methyl-1-phenylbutane	Benzenoids	3.59E-05	1.02E-04	2.092	0.011	0.352	0.791
3-Carbamoyl-2-phenylpropionaldehyde	Benzenoids	1.94E-05	5.25E-05	2.068	0.013	0.369	0.75
L-Pyridosine	Organic acids and derivatives	8.16E-06	1.77E-05	1.76	0.010	0.461	0.75
N-Methylcalystegine B2	Alkaloids and derivatives	3.68E-05	5.91E-05	1.449	0.030	0.623	0.742
Aesculin	Phenylpropanoids and polyketides	1.80E-05	3.90E-05	1.701	0.003	0.461	0.74
Betonicine	Organic acids and derivatives	3.36E-05	6.89E-05	1.88	0.002	0.488	0.721
Prolylphenylalanine	Organic acids and derivatives	2.39E-05	3.11E-05	1.68	0.020	0.769	0.708
PC(20:1(11Z)/20:1(11Z))	Lipids and lipid-like molecules	1.38E-06	1.27E-05	2.318	0.001	0.109	0.705
Valyl-Tyrosine	Organic acids and derivatives	3.02E-05	5.20E-05	1.672	0.033	0.581	0.704
p-Anisic acid	Benzenoids	7.73E-05	3.97E-04	1.955	0.013	0.195	0.7
Tyrosyl-Serine	Organic acids and derivatives	2.38E-05	4.02E-05	1.441	0.036	0.592	0.695
Harderoporphyrin	Organoheterocyclic compounds	2.97E-06	8.05E-06	1.972	<0.001	0.369	0.668
Caffeine	Organoheterocyclic compounds	1.61E-04	4.15E-04	2.051	0.011	0.386	0.654
4,8 Dimethylnonanoyl carnitine	Lipids and lipid-like molecules	8.20E-06	1.95E-05	1.997	0.005	0.42	0.644
D-Glucurono-6,3-lactone	Organoheterocyclic compounds	4.32E-05	1.70E-04	1.923	0.018	0.254	0.644
Progesterone	Lipids and lipid-like molecules	7.40E-05	1.60E-04	1.554	0.039	0.462	0.636
Ascorbic acid	Organoheterocyclic compounds	2.63E-05	7.26E-05	1.747	0.036	0.362	0.627
Myrigalone H	Phenylpropanoids and polyketides	2.02E-05	3.43E-05	1.66	0.033	0.59	0.615
Pyrophaeophorbide a	Organoheterocyclic compounds	7.42E-05	1.90E-04	1.646	0.034	0.391	0.593
Threonic acid	Organic oxygen compounds	6.77E-05	3.50E-04	2.04	<0.001	0.193	0.577
1,2,3,4-Tetrahydro-b-carboline-1,3-dicarboxylic acid	Alkaloids and derivatives	7.03E-06	1.37E-05	1.899	0.016	0.512	0.568
Simvastatin	Organoheterocyclic compounds	6.64E-06	4.89E-05	2.265	0.001	0.136	0.55
Glycerol tripropanoate	Lipids and lipid-like molecules	1.44E-04	2.30E-04	1.4	0.047	0.628	0.541
Saccharin	Organoheterocyclic compounds	3.82E-06	3.98E-05	2.151	0.003	0.096	0.536

PE(15:0/14:0)	Lipids and lipid-like molecules	1.23E-04	1.80E-04	1.481	0.045	0.681	0.512
L-cis-3-Amino-2-pyrrolidinecarboxylic acid	Organic acids and derivatives	2.53E-05	5.79E-05	1.853	0.025	0.437	0.488
PE(16:0/14:0)	Lipids and lipid-like molecules	3.15E-04	4.83E-04	1.517	0.029	0.651	0.479
PI(20:2(11Z,14Z)/18:2(9Z,12Z))	Lipids and lipid-like molecules	3.12E-05	7.22E-05	1.584	0.024	0.433	0.447
2-Oxoarginine	Organic acids and derivatives	3.35E-05	6.30E-05	1.663	0.025	0.532	0.445
L-Histidine	Organic acids and derivatives	3.18E-04	4.54E-04	1.653	0.020	0.701	0.44
(3beta,17alpha,23S)-17,23-Epoxy-3,28,29-trihydroxy-27-norlanost-8-en-24-one	Lipids and lipid-like molecules	2.71E-05	4.82E-05	1.625	0.025	0.561	0.436
Norfluoxetine glucuronide	Organic oxygen compounds	9.94E-06	1.59E-05	1.48	0.042	0.627	0.432
Homovanillic acid	Benzenoids	4.56E-05	1.29E-04	1.951	0.002	0.353	0.416
Catechin	Phenylpropanoids and polyketides	7.23E-05	1.89E-04	1.949	0.002	0.383	0.403

The differences were analyzed by using Student’s *t* test. VIP stands for the variable importance for the projection in the OPLS-DA analyses.

MS2 score (between 0 and 1) stands for the fragmentation score based on MS2 library searches.

Table S4. The gut metabolites significantly decreased after one-month of oral SrR.

Name	Super Class	Mean of OVX	Mean of OVX_Sr	VIP	*P* Value	Fold change	MS2 score
Ricinoleic acid	Lipids and lipid-like molecules	1.31E-03	6.82E-04	1.536	0.028	1.918	0.975
5'-Methylthioadenosine	Nucleosides, nucleotides, and analogues	3.12E-04	1.56E-04	1.381	0.034	1.997	0.969
Pyridoxine	Organoheterocyclic compounds	8.01E-04	3.43E-04	1.603	0.030	2.335	0.959
Alpha-dimorphecolic acid	Lipids and lipid-like molecules	7.02E-04	3.30E-04	1.774	0.005	2.130	0.941
Erucic acid	Lipids and lipid-like molecules	3.31E-04	1.14E-04	1.812	0.001	2.910	0.932
Indole-3-carboxylic acid	Organoheterocyclic compounds	7.17E-04	1.41E-04	2.062	0.000	5.104	0.931
4-Pyridoxic acid	Organoheterocyclic compounds	1.80E-03	1.17E-03	1.521	0.010	1.540	0.927
Unknown 370	Lipids and lipid-like molecules	6.14E-06	4.37E-06	1.485	0.022	1.405	0.895
9,10-DHOME	Lipids and lipid-like molecules	2.53E-04	1.08E-04	1.404	0.017	2.351	0.892
5-Methyldeoxycytidine	Nucleosides, nucleotides, and analogues	1.83E-03	1.14E-03	1.530	0.009	1.602	0.884
Geranylgeranyl-PP	Lipids and lipid-like molecules	2.77E-05	1.81E-05	1.402	0.014	1.528	0.880
N-Acetylvanilalanine	Organic acids and derivatives	6.66E-05	2.81E-05	1.937	0.000	2.372	0.864
Asparaginyl-Hydroxyproline	Organic acids and derivatives	5.51E-04	3.86E-04	1.538	0.020	1.428	0.864
LysoPE(0:0/22:6(4Z,7Z,10Z,13Z,16Z,19Z))	Lipids and lipid-like molecules	2.84E-06	1.54E-06	1.549	0.036	1.846	0.854
Pyrrole-2-carboxylic acid	Organoheterocyclic compounds	5.43E-05	3.40E-05	1.471	0.011	1.596	0.853
Pipercide	Organoheterocyclic compounds	1.28E-05	3.07E-06	1.504	<0.001	4.175	0.847
1-(Hydroxymethyl)-5,5-dimethyl-2,4-imidazolidinedione	Organoheterocyclic compounds	1.87E-04	2.94E-05	2.375	<0.001	6.349	0.847
Adenosine	Nucleosides, nucleotides, and analogues	2.49E-05	7.73E-06	1.659	0.038	3.225	0.845
Tiglylglycine	Organic acids and derivatives	8.12E-04	3.46E-04	1.911	<0.001	2.346	0.842
[4]-Gingerdiol 3,5-diacetate	Benzenoids	8.03E-05	5.52E-05	1.303	0.037	1.454	0.838

Pectachol	Phenylpropanoids and polyketides	4.96E-06	7.71E-07	2.250	0.002	6.437	0.834
Ginsenoyne N	Organoheterocyclic compounds	8.04E-06	3.27E-06	2.143	0.001	2.458	0.831
Hydroxyprolyl-Valine	Organic acids and derivatives	2.69E-05	1.72E-05	1.853	0.004	1.564	0.816
Valyl-Lysine	Organic acids and derivatives	1.59E-04	5.47E-05	1.620	0.022	2.898	0.815
2-Keto-6-acetamidocaproate	Organic acids and derivatives	1.16E-03	2.97E-04	2.242	<0.001	3.905	0.802
2-Diethylaminoethanol	Organic nitrogen compounds	3.40E-03	5.11E-05	2.407	<0.001	66.499	0.785
Indole-3-carbinol	Organoheterocyclic compounds	8.27E-05	2.59E-05	1.305	0.050	3.194	0.783
Pterolactam	Organoheterocyclic compounds	9.50E-05	6.45E-05	1.559	0.024	1.472	0.783
N-Cyclopropyl-trans-2-cis-6-nonadienamide	Lipids and lipid-like molecules	8.42E-05	4.49E-05	2.059	<0.001	1.875	0.778
Heterotropan	Lignans, neolignans and related compounds	6.47E-06	3.20E-06	1.314	0.041	2.021	0.769
LysoPA(0:0/18:0)	Lipids and lipid-like molecules	8.28E-06	4.66E-06	1.130	0.048	1.777	0.755
1-Oleoylglycerophosphoinositol	Lipids and lipid-like molecules	3.24E-06	6.19E-07	1.480	0.032	5.238	0.742
Glucoemodin	——	9.01E-06	3.10E-06	1.758	0.036	2.906	0.730
Lysyl-Valine	Organic acids and derivatives	3.21E-04	1.42E-04	1.663	0.022	2.265	0.730
Lanthionine ketimine	Organic acids and derivatives	1.93E-05	1.04E-05	1.654	0.009	1.845	0.725
2-Furoylglycine	Organic acids and derivatives	6.07E-05	4.34E-05	1.243	0.041	1.396	0.712
4,8,12,15-Octadecatetraenoic acid	Lipids and lipid-like molecules	1.09E-04	6.81E-05	1.866	0.001	1.606	0.697
9-HOTE	Lipids and lipid-like molecules	1.94E-04	1.10E-04	1.849	0.006	1.767	0.683
L-Histidinol	Organic nitrogen compounds	3.21E-05	1.56E-05	2.016	<0.001	2.064	0.676
Triethyl citrate	Organic acids and derivatives	4.20E-05	9.10E-06	2.109	0.013	4.623	0.671
3-Indolebutyric acid	Organoheterocyclic compounds	8.10E-05	2.02E-05	2.008	0.000	4.014	0.649
Licoagrochalcone B	Phenylpropanoids and polyketides	5.85E-05	4.22E-05	1.416	0.041	1.388	0.646
(2'E,4'Z,8E)-Colneleic acid	Lipids and lipid-like molecules	2.02E-04	1.01E-04	1.702	0.007	2.000	0.631
Dieporeticenin	Lipids and lipid-like molecules	1.42E-05	7.42E-06	1.778	0.008	1.916	0.624

Dihydrojasmonic acid	Lipids and lipid-like molecules	8.34E-04	2.65E-04	1.755	0.003	3.153	0.617
Gentisic acid	Benzenoids	2.82E-04	1.33E-04	1.596	0.031	2.120	0.606
beta-Cryptoxanthin	Lipids and lipid-like molecules	1.04E-04	6.80E-05	1.347	0.022	1.529	0.601
Iprobenfos	Benzenoids	8.72E-05	5.10E-05	1.616	0.012	1.708	0.597
Deoxycytidine	Nucleosides, nucleotides, and analogues	6.20E-04	2.92E-04	1.895	0.001	2.119	0.595
2-Amino-3-methylbenzoate	Benzenoids	1.65E-04	5.81E-05	2.008	<0.001	2.844	0.589
9-HODE	Lipids and lipid-like molecules	1.87E-03	8.35E-04	1.096	0.028	2.243	0.565
m-Chlorohippuric acid	Benzenoids	4.50E-05	2.03E-05	1.956	<0.001	2.211	0.559
Dihydrozeatin	Organoheterocyclic compounds	4.18E-05	2.56E-05	1.446	0.025	1.634	0.551
PE(22:4(7Z,10Z,13Z,16Z)/14:1(9Z))	Lipids and lipid-like molecules	2.50E-05	1.46E-05	1.571	0.014	1.710	0.536
(3R)-3,4-Dihydroxy-3-(hydroxymethyl)butanenitrile 4-glucoside	Lipids and lipid-like molecules	5.05E-05	1.88E-05	1.120	0.022	2.691	0.529
9-Methylxanthine	Organoheterocyclic compounds	1.27E-04	2.44E-05	2.126	<0.001	5.199	0.520
Saccharopine	Organic acids and derivatives	9.12E-06	4.30E-06	1.792	0.003	2.124	0.519
Orotic acid	Organoheterocyclic compounds	4.95E-05	2.76E-05	1.372	0.011	1.790	0.498
1,4,5-Naphthalenetriol	Benzenoids	2.33E-04	1.28E-04	1.525	0.026	1.824	0.454
Cystathionine ketimine	Organic acids and derivatives	2.69E-05	1.23E-05	1.577	0.002	2.193	0.448
Levocetirizine	Benzenoids	2.72E-05	1.16E-05	1.962	0.003	2.351	0.430

The differences were analyzed by using Student's *t* test. VIP stands for the variable importance for the projection in the OPLS-DA analyses. MS2 score (between 0 and 1) stands for the fragmentation score based on MS2 library searches.

Table S5. The Spearman's relationship between the relative abundance of *R. albus* and BMD.

	r	*P* value
BMD of the whole body	0.7795	0.0014
BMD of the body without head	0.7474	0.0026
BMD of the hindquarters	0.6941	0.0061
